# Risk Factors for Contralateral Occult Carcinoma in Patients With Unilateral Papillary Thyroid Carcinoma: A Retrospective Study and Meta-Analysis

**DOI:** 10.3389/fendo.2021.675643

**Published:** 2021-07-12

**Authors:** Fan Zhang, Boyuan Zheng, Xiaohui Yu, Xichang Wang, Shiwei Wang, Weiping Teng

**Affiliations:** ^1^ Department of Endocrinology and Metabolism, Institute of Endocrinology, National Health Commission Key Laboratory of Diagnosis and Treatment of Thyroid Diseases, The First Hospital of China Medical University, Shenyang, China; ^2^ Department of Thyroid Surgery, The First Hospital of China Medical University, Shenyang, China

**Keywords:** contralateral, meta-analysis, occult, papillary thyroid carcinoma, risk factors

## Abstract

**Background:**

Bilateral lesions are common in papillary thyroid carcinoma (PTC). For patients with unilateral PTC, occult carcinoma that is not detected preoperatively, but pathologically after surgery, might remain in the contralateral lobe. In this situation, inadequate surgical extent could cause relapse and even lead to re-operation. Here, we explore the frequency and investigate the risk factors of contralateral occult PTC in unilateral PTC through a retrospective study conducted by our team and published articles online, respectively.

**Methods:**

We collected the patients’ clinical data in our hospital, whose cancer was determined to be confined to the unilateral lobe by preoperative image examination (N = 204). These patients underwent initially total or near-total thyroidectomy and included their clinical data in the meta-analysis. We searched related literature in the PubMed, Embase, MEDLINE, Cochrane, and Web of Science databases until December 7, 2020, in order to perform a meta-analysis. The relevant articles were examined and the eligible studies were included to assess the association between clinicopathologic factors and contralateral occult PTC.

**Results:**

The meta-analysis included nine studies (involving 4347 patients). Of these, eight studies were from the databases, and one study was our retrospective data. The meta-analysis showed that the prevalence of contralateral occult PTC was 26.6% in all patients. A tumor size > 1 cm, ipsilateral multifocality, contralateral benign nodule, and central lymph node metastasis were significantly associated with contralateral occult PTC. In contrast, sex, age, ETE, capsular invasion, *BRAF* mutation, Hashimoto thyroiditis, and lateral lymph node metastasis were insignificantly associated with contralateral occult PTC.

**Conclusion:**

The meta-analysis identified a tumor size > 1 cm, ipsilateral multifocality, contralateral benign nodule, and CLNM as being significant risk factors for contralateral occult PTC. These findings may guide the extent of surgery in unilateral PTC patients.

## Introduction

Thyroid carcinoma is the most frequent endocrine malignancy, accounting for approximately 2.9% of all newly diagnosed cancer (1). Its incidence has increased from 5.7 to 14.7 per 100,000 people over the past two decades (2). Papillary thyroid carcinoma (PTC), medullary thyroid carcinoma, follicular thyroid carcinoma, and anaplastic thyroid carcinoma are the four main types of thyroid carcinoma. PTC is the most common thyroid cancer type, accounting for approximately 80.0% of malignant thyroid tumors (3). In addition, papillary thyroid microcarcinoma (PTMC) is classified to PTC. According to the histological classification of thyroid tumors by the World Health Organization, PTMC is defined as tumors with a maximum size of 10 mm or smaller. PTC has an excellent prognosis, with the main treatment being surgical resection. The overall survival rate has remained between 90% and 96% in recent decades (4, 5).

Despite advances in the understanding of the underlying biological characteristics of PTC and the development of evidence-based guidelines for its treatment, the adequate extent of surgical management (lobectomy *vs*. total thyroidectomy) of PTC is still a matter of debate. Neck ultrasound (US) and US-guided fine-needle aspiration (FNA) are currently used in decision making for the surgical management of PTC. However, despite that high-resolution US can detect foci as small as 2 mm (6), smaller tumors remain undetected. Occult carcinoma is defined as a tumor that is not detected preoperatively but pathologically after surgery. As reported, PTC frequently occurs as multifocal or bilateral lesions, with the prevalence of multifocality ranging from 20.0% to 36.1% (7–9). The 2015 American Thyroid Association Guidelines (10) endorse thyroid lobectomy (TL) as an initial surgical approach for low risk, small- to medium-sized (T1-T2), N0 PTC in the absence of an extrathyroidal extension (ETE). For PTCs detected preoperatively in bilateral lobes, there is no controversy in performing total thyroidectomy (TT). Contralateral occult PTC is a specific subtype of bilateral multifocal PTC. It has been reported that the rate of occult PTC in the contralateral lobe ranges from 12% to 40% (11–13). For contralateral occult PTC, TL may be insufficient and could cause relapse and even lead to re-operation, which brings higher risks. Therefore, identifying risk factors of contralateral occult PTC could help surgeons to determine the optimal scope of surgery, but there is a limited number of studies focusing on risk factors of contralateral occult PTC. In this study, we used our retrospective research data and reviewed all related literature on the website that conducted this meta-analysis, aiming to estimate the risk factors for contralateral occult carcinoma in patients with unilateral PTC.

## Materials and Methods

We collected the clinical data of patients with PTC treated in the First Hospital of China Medical University (Shenyang, China) from January 2016 to January 2021. Data collected included age, sex, thyroid ultrasound report, operation record, diagnosis, and pathology report. The inclusion criteria were as follows: PTC confined to the only unilateral lobe by preoperative image examinations or fine-needle aspiration cytology; patients who underwent initially total or near-total thyroidectomy; and postoperative pathological examination confirming the unilateral lesion was PTC. Also, the exclusion criteria were as follows: the patients had a suspicious lesion on the contralateral lobe; PTC located in the isthmus. Finally, 204 patients were included in our study, in which 27 patients were with contralateral occult PTC (confirmed by pathological examination). These clinical data were included in the meta-analysis.

Our meta-analysis was conducted according to the guidelines proposed by the preferred reporting items for systematic review and meta-analyses statement (14).

### Search Strategy

An electronic search of the PubMed, Embase, MEDLINE, Cochrane, and Web of Science databases was performed to identify relevant articles until December 7, 2020. We used the following search terms in “all fields”: ((occult) AND (contralateral OR bilateral), AND (papillary thyroid) AND (carcinoma OR cancer OR tumor OR neoplasm)). Besides, a manual review of the references from the included studies was performed to identify additional relevant articles. Two authors independently performed the selection process, and the discrepancies were resolved by discussion. If multiple published studies illustrated the same population, we extracted the most complete or recent one.

### Selection Criteria

We included studies that met the following criteria:

1. Prospective or retrospective original studies.
2. PTC confined to only the unilateral lobe by preoperative image examinations or FNA.
3. Patients had initially undergone total or near-total thyroidectomy.
4. Pathologic examination confirming the presence of contralateral occult PTC.
5. Risk factors of contralateral occult PTC were available for extraction to calculate the pooled odds ratio (OR).

We excluded studies by the following criteria:

1. Reviews, case reports, letters to the editor, and conference abstracts.
2. Studies published in languages other than English and Chinese.
3. Studies enrolled patients with follicular thyroid carcinoma, medullary thyroid carcinoma, or anaplastic carcinoma.
4. The patients had a suspicious lesion on the contralateral lobe.
5. PTC located in the isthmus.
6. Lacked complete clinical data.

## Data Extraction and Quality Assessment

Each eligible study was extracted by two independent reviewers. Disagreements were resolved by consensus or adjudication of the senior authors. We extracted the following data from the included studies: (1) study characteristics: first author, publication year, country of study, study design, study population (PTC or PTMC), number of patients, and surgical extent; (2) clinicopathologic factors for contralateral occult carcinoma: sex, age, tumor size, ipsilateral multifocality, ETE, capsular invasion, *BRAF* mutation, Hashimoto thyroiditis (HT), presence of contralateral benign nodule, central lymph node metastasis (CLNM), and lateral lymph node metastasis (LLNM).

The included studies were estimated using the Newcastle-Ottawa scale (15), which allows evaluation of cohort studies by a total of eight items of three major parts, including the study population selection, comparability, and the result. A score of 0 to 9 stars was used to assess each study. A study achieving more than six stars was considered a high-quality study. All quality assessments were performed by two independent reviewers.

## Statistical Analysis

The retrospective research data statistical analyses were performed using the SPSS v 26.0 software (Chicago, USA). The continuous variables are expressed as a mean ± standard deviation (SD). Univariate analysis for comparisons between patient groups was performed using Pearson’s chi-square test or Fisher’s exact test. Variables with a p < 0.05 in the univariate analysis were included in the multivariable analysis. Logistic regression analysis was performed to assess the risk factors of contralateral occult carcinoma.

We performed the meta-analysis by Revman Manager 5.0 (Cochrane Collaboration, Oxford, UK). The OR was used to compare dichotomous variables. All results were estimated with 95% confidence intervals (CIs). A P-value < 0.05 was considered statistically significant. Heterogeneity was examined by using the Q-test and I^2^ statistic. When p > 0.1 and I^2^ < 50%, a fixed-effect model was used; otherwise, a random-effects model was applied. Possible publication bias was tested using Begg’s funnel plot.

## Results

### Retrospective Study

#### Patients and Tumor Characteristics

After screening the medical records, 204 patients met the criteria and were included in the study. Of these patients, 47 (23.0%) were men, and 157 (77.0%) were women. The average age was 42.5 years, and the age range was 22 to 67 years. Briefly, 172 (84.3%) patients were younger than 55 years, and 32 (15.7%) were 55 years and older. All patients underwent surgical treatment. The average tumor size was 1.28 cm, and the size range was 0.32 to 5.80 cm. In 93 (45.6%) patients, the tumor size was ≤ 1 cm; and in 111 (54.4%) patients, the tumor size was > 1 cm. For the patients included, 27 (13.2%) had contralateral occult carcinoma and 177 (13.2%) did not; 21 (10.3%) patients had ETE and 183 (89.7%) patients did not; 65 (31.9%) patients had central lymph node metastasis, 40 (19.6%) patients had lateral lymph node metastasis, and 99 (48.5%) patients were without lymph node metastasis (**Table 1**).

**Table 1:** Associations between clinicopathological characteristics and contralateral occult carcinoma in PTC patients.

Variables	Overall (N=204)	Contralateral occult	Non-contralateral occult lesion	P value	OR	95% CI	P value
		N = 27 (13.2%)	N = 177 (86.8%)				
Sex							
Male	47 (23.0)	5 (10.6)	42 (89.4)	0.549			
Female	157 (77.0)	22 (14.0)	135 (86.0)				
Age (Y)	42.51 ± 10.42	42.85 ± 9.12	42.46 ± 10.63				
<55	172 (84.3)	23 (13.4)	149 (86.6)	0.894			
≥55	32 (15.7)	4 (12.5)	28 (87.5)				
Tumor size (cm)	1.28 ± 0.77	2.00 ± 1.29	1.14 ± 0.60				
≤1	94 (46.1)	6 (6.4)	88 (93.6)	0.008	1		
>1	110 (53.9)	21 (19.1)	89 (80.9)		3.461	1.333–8.984	0.011
ETE							
Absence	183 (89.7)	16 (8.7)	167 (91.3)	<0.001	1		
Presence	21 (10.3)	11 (52.4)	10 (47.6)		11.481	4.231–31.154	<0.001
LNM							
Without LNM	99 (48.5)	5 (5.1)	94 (94.9)	0.004	1		
CLNM	65 (31.9)	14 (21.5)	51 (78.5)		2.086	1.246–3.495	0.005
LLNM	40 (19.6)	8 (20.0)	32 (80.0)		6.765	1.877–24.380	0.003

PTC, papillary thyroid carcinoma; Y, year; ETE, extrathyroidal extension; LNM, lymph node metastasis; CLNM, central lymph node metastasis; LLNM, lateral lymph node metastasis; OR, odds ratio; 95% CI, 95% confidence interval.

#### Clinicopathological Factors Associated With Contralateral Occult Carcinoma

Among the 27 patients with occult lesions in the contralateral gland, 5 (18.5%) were men and 22 (81.5%) were women. Twenty-three (85.2%) patients were younger than 55 years and four (12.5%) patients were 55 years and older. The mean tumor size of the contralateral occult lesion was 2.00 ± 1.29, and the non-contralateral occult lesion was 1.14 ± 0.60. Six (22.2%) patients had a tumor size ≤ 1 cm, and 21 (77.7%) patients had a tumor size > 1 cm. Contralateral occult carcinoma presented with a significant association with tumor size and the presence of ETE, CLNM, and LLNM by univariate analysis (all p < 0.05). All of these factors were included in the multivariate analysis, which showed that a tumor size >1 cm (OR = 3.461, 95% CI = 1.333–8.984, p = 0.011) and the presence of ETE (OR = 11.481, 95% CI = 4.231–31.154, p < 0.001), CLNM (OR = 2.086, 95% CI = 1.246–3.495, p = 0.005), and LLNM (OR = 6.765, 95% CI = 1.877–24.380, p = 0.003) were independent predictors of contralateral occult carcinoma (**Table 1**).

### Meta-Analysis

**Figure 1:**
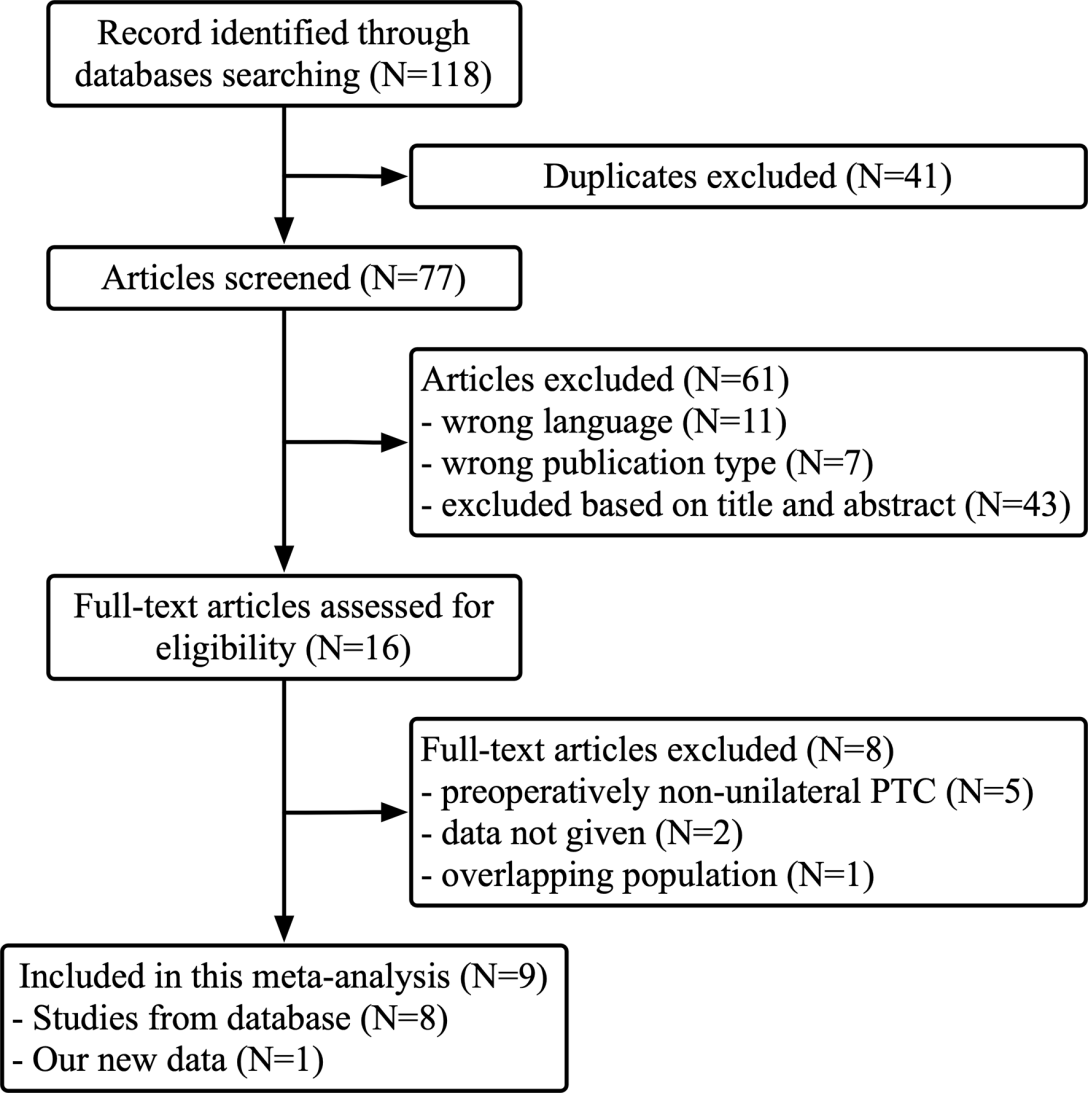
Flow chart of the study selection process.

After searching the databases, 118 studies were initially found, and 77 articles were determined to be non-overlapping articles. Eleven articles were excluded because of language, seven studies were excluded because they were case reports, and 43 studies were excluded because the title or abstract was not applicable. The remaining 16 articles were subjected to a full-text evaluation. However, patients in the five studies were non-unilateral PTC preoperatively, data could not be obtained in two studies, and one study had an overlapping population. A flowchart with details is displayed in **Figure 1**. Finally, a total of nine studies were included in the meta-analysis with our retrospective research data included. **Table 2** shows the baseline characteristics and quality scores of the nine studies included, which consisted of two prospective and seven retrospective studies with a total of 4347 patients enrolled. Eight studies were conducted in China and one in South Korea. All the studies enrolled patients who underwent TT or NT for the treatment of PTC confined to the unilateral lobe, without suspicious carcinoma lesion in the contralateral lobe by preoperative image examinations or FNA. Contralateral occult PTC was defined as a tumor lesion detected by pathology postoperatively in the contralateral lobe rather than detected by preoperative examinations. The prevalence of contralateral occult PTC in each study was available and ranged from 11.95% to 46.9% (median 16.7%). Among the 4,347 patients enrolled, contralateral occult PTC was reported in 1,150 patients, and the prevalence was 26.6%. According to Newcastle-Ottawa Scale (NOS), three studies were rewarded 6 stars, and six studies were rewarded 7 stars. The average award of the nine studies was 6.7 stars on a scale of 0 to 9. All studies were considered adequate for meta-analysis.

**Table 2:** Characteristics of the nine included studies.

Author	Year	Country	Study Period	Study Design	Study Population	Case	Surgical Extent	Prevalence of contralateral occult PTC (%)	Quality assessment
Chen XH (12)	2020	China	2014/1–2017/9	Retrospective	PTC	921	TT/NT+CLND/CLND+LLND	16.7	6
Feng JW (16)	2020	China	2011/1–2018/1	Retrospective	PTC	552	TT+CLND/CLND+LLND	16.1	7
Gu JL (17)	2020	China	2012/9–2014/9	Retrospective	PTC	300	TT+CLND/CLND+LLND	18.3	7
Koo BS (18)	2010	Korea	2005/3–2009/3	Retrospective	PTMC	132	TT+CLND	16.7	7
Lv T (19)	2018	China	2014/1–2016/12	Retrospective	PTC	1442	TT+CLND	46.9	7
Wan HF (13)	2015	China	2011/1–2013/12	Prospective	PTMC	89	TT+ selective LND	40.4	7
Wang N (11)	2020	China	2016/5–2018/6	Retrospective	PTC	586	TT+ cervical LND	11.95	6
Zhou YL (20)	2012	China	2010/11–2011/11	Prospective	PTMC	100	TT+CLND	20	6
Our new data	2021	China	2016/1–2021/1	Retrospective	PTC	204	TT+CLND/CLND+LLND	13.2	7

PTC, papillary thyroid carcinoma; PTMC, papillary thyroid microcarcinoma; TT, total thyroidectomy; NT, near-total thyroidectomy; CLND, central lymph node dissection; LLND, lateral lymph node dissection; LND, lymph node dissection.

### Sex

There were 204 patients who met the criteria and were included in this retrospective study. Of these patients, 47 (23.0%) were men and 157 (77.0%) were women. There were 27 (13.2%) patients who had contralateral occult carcinoma and 177 (13.2%) did not; among the 27 patients with contralateral occult PTC, 5 (18.5%) were men, and 22 (81.5%) were women, sex had nothing to do with whether patients had contralateral occult PTC (**Table 1**).

**Figure 2:**
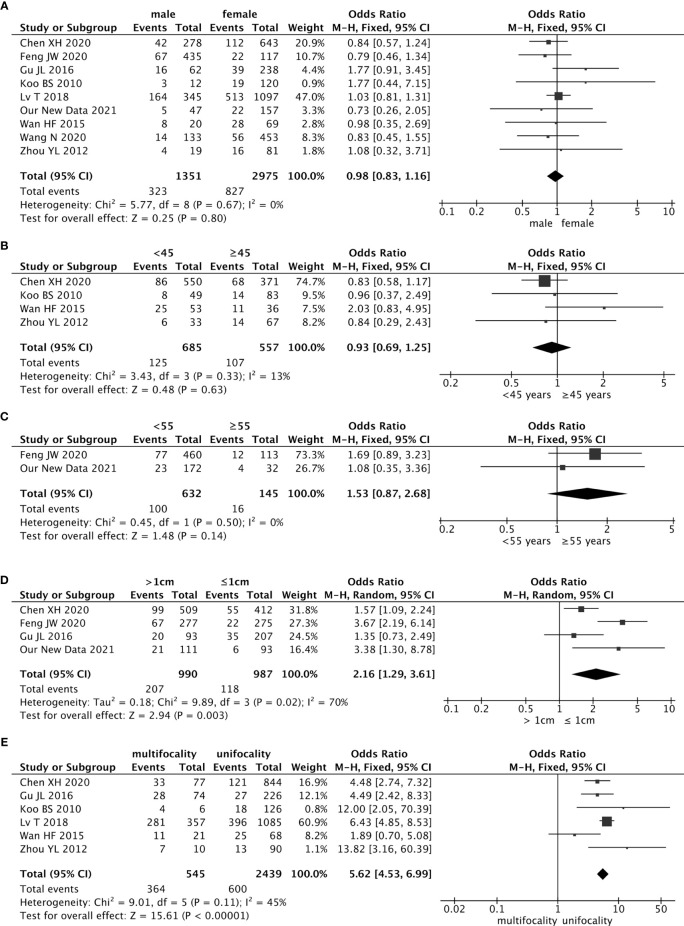
Forest plots of the association between contralateral occult PTC (papillary thyroid carcinoma) and all risk factors: **(A)** Sex was not associated with contralateral occult PTC (pooled OR = 0.98, 95% CI = 0.83–1.16, p = 0.80); **(B)** The risk of age (cut-off value was 55) was not associated with contralateral occult PTC (pooled OR = 1.53, 95% CI = 0.87–2.68, p = 0.14); **(C)** The risk of age (cut-off value was 45) was not associated with contralateral occult PTC (pooled OR = 0.93, 95% CI = 0.69–1.25, p = 0.63); **(D)** The risk of contralateral occult PTC was significantly higher in patients with a tumor > 1 cm than patients with a tumor ≤ 1 cm (pooled OR = 2.16, 95% CI = 1.1.29–3.61, p = 0.003); **(E)** Compared with patients without ipsilateral multifocality, patients with ipsilateral multifocality had a higher prevalence of contralateral occult PTC (pooled OR = 5.62, 95% CI = 4.53–6.99, p < 0.00001).

Nine studies reported the relationship between the risk of contralateral occult PTC and sex, including our retrospective study. The pooled analysis revealed that sex was not associated with contralateral occult PTC (pooled OR = 0.98, 95% CI – 0.83–1.16, p = 0.80) (**Figure 2A**).

### Age

Age is a critical factor in outcomes for patients with well-differentiated thyroid cancer. Forty-five years was used as the cutoff value in staging before 2016. In four studies, the cutoff value for age was 45 years, and the pooled analysis revealed that 45 years as a cutoff value for age was not associated with the contralateral occult PTC (pooled OR = 0.93, 95% CI = 0.69–1.25, p = 0.33) (**Figure 2B**).

However, there is increasing evidence to suggest that regarded 45 years as the cutoff value for age may be too low. The newest guideline in the AJCC/UICC staging system changed the cutoff value for age from 45 years to 55 years in 2017. In our retrospective study, the patients’ average age was 42.5 years, and the age range was 22 to 67 years. Briefly, 172 (84.3%) patients were younger than 55 years, and 32 (15.7%) patients were 55 years and older. In the 27 patients who had occult lesions in the contralateral gland, 23 patients (85.2%) were younger than 55 years, and 4 patients (12.5%) were 55 years or older. There were no differences on whether which patients had contralateral occult PTC between the two groups younger than 55 years and 55 years or older (**Table 1**). The results of the meta-analysis showed that the age cutoff value of 55 years was not associated with the contralateral occult PTC (pooled OR = 1.53, 95% CI = 0.87–2.68, p = 0.50) (**Figure 2C**).

### Tumor Size

In our retrospective study of 204 included PTC patients, the average tumor size was 1.28 cm, and the size range was 0.32 to 5.80 cm. In 93 (45.6%) patients, the tumor size was ≤ 1 cm, and for the other 111 (54.4%) patients, the tumor size was > 1 cm. However, in the contralateral occult PTC group, the mean tumor size of the lesion was 2.00 ± 1.29; and in the non-contralateral occult group, this value was 1.14 ± 0.60. In the contralateral occult PTC group, there were 6 (22.2%) patients whose tumor size was ≤ 1 cm, and 21 (77.7%) patients whose tumor size was > 1 cm. The results of the univariate analysis showed that the presence of contralateral occult PTC had a significant association with tumor size (p<0.05). Also, the multivariable analysis showed that tumor size >1 cm (OR = 3.461, 95% CI = 1.333–8.984, p = 0.011) was an independent predictor of contralateral occult PTC (**Table 1**).

There were four studies (included our study) that estimated the effect of tumor size on contralateral occult PTC using the cutoff value of 1.0 cm. Findings from the pooled analysis suggested that the risk of contralateral occult PTC was significantly higher in patients with a tumor size > 1 cm than patients with a tumor size ≤ 1 cm (pooled OR = 2.16, 95% CI = 1.29–3.61, p = 0.003) (**Figure 2D**).

### Ipsilateral Multifocality

There were six studies that addressed the association between the risk of contralateral occult PTC and ipsilateral multifocality. Because of the low heterogeneity among the studies (I^2^ = 45%, p = 0.11), a fixed-effects model was used to analyze the data. Compared with patients without ipsilateral multifocality, patients with ipsilateral multifocality had a higher prevalence of contralateral occult PTC (pooled OR = 5.62, 95% CI = 4.53–6.99, p < 0.00001) (**Figure 2E**).

### Extrathyroidal Extension (ETE)

In the 204 PTC patients included in this retrospective study, 21 (10.3%) patients had ETE and 183 patients (89.7%) did not. However, in the contralateral occult PTC group, there were 11 (40.7%) patients with ETE, the other 16 patients (59.3) did not. The results of the univariate analysis showed that the presence of contralateral occult PTC had a significant association with ETE (p<0.05). Also, the multivariable analysis showed that the presence of ETE (OR = 11.481, 95% CI = 4.231 (40.7%)31.154, p < 0.001) was an independent predictor of contralateral occult PTC (**Table 1**).

**Figure 3:**
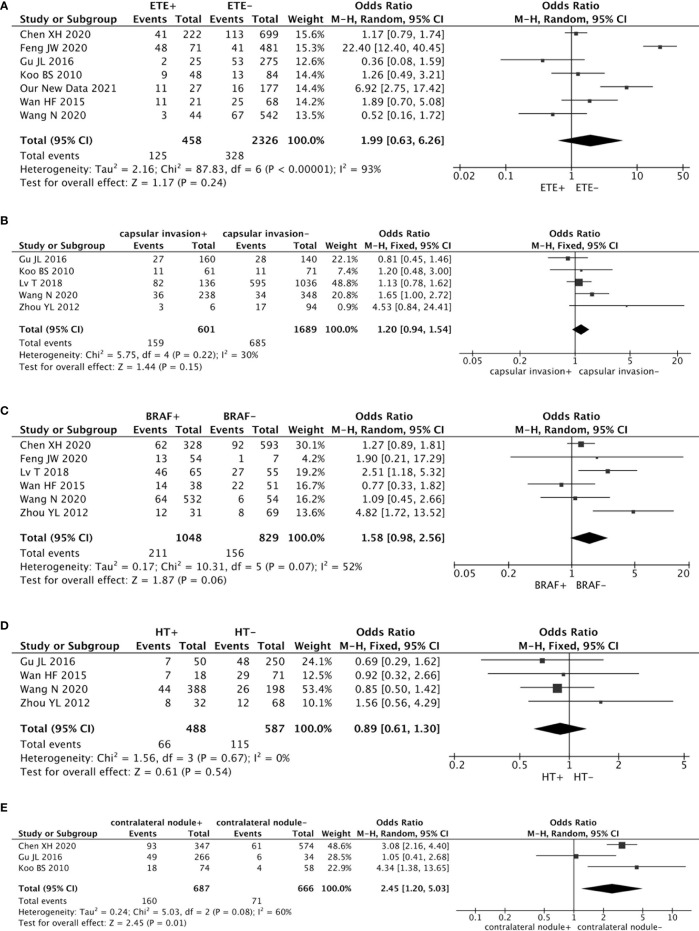
Forest plots of the association between contralateral occult PTC and all factors: **(A)** There was no significant association between ETE and contralateral occult PTC (pooled OR = 1.99, 95% CI = 0.63–6.26, p = 0.24); **(B)** The risk of capsular invasion was not associated with contralateral occult PTC (pooled OR = 1.20, 95% CI = 0.94–1.54, p = 0.15); **(C)** There was no significant association between a *BRAF* mutation and contralateral occult PTC (pooled OR = 1.58, 95% CI = 0.98–2.56, p = 0.06); **(D)** The risk of HT was not associated with contralateral occult PTC (pooled OR = 0.89, 95% CI = 0.61–1.30, p = 0.54); **(E)** Compared with patients without a contralateral benign nodule, patients with a contralateral benign nodule had a higher prevalence of contralateral occult PTC (pooled OR = 2.45, 95% CI = 1.20–5.03, p = 0.01).

Seven studies analyzed the impact of ETE on contralateral occult PTC (including our new data). This pooled analysis suggested no significant association between ETE and contralateral occult PTC (pooled OR = 1.99, 95% CI = 0.63–6.26, p = 0.24) (**Figure 3A**).

### Capsular Invasion

Five studies addressed the association between the risk of contralateral occult PTC and capsular invasion. This risk factor showed low heterogeneity among the studies (I^2^ = 30%, p = 0.22). Results from the meta-analyses exhibited that the risk of capsular invasion was not associated with contralateral occult PTC (pooled OR = 1.20, 95% CI = 0.94–1.54, p = 0.15) (**Figure 3B**).

### *BRAF* Mutation

Six studies analyzed the impact of a *BRAF* mutation on contralateral occult PTC. The heterogeneity was 52% (p = 0.07). This pooled analysis suggested no significant association between a *BRAF* mutation and contralateral occult PTC (pooled OR = 1.58, 95% CI = 0.98–2.56, p = 0.06) (**Figure 3C**).

### Hashimoto Thyroiditis (HT)

An association between the risk of contralateral occult PTC and HT was investigated in four studies. Because no statistically significant heterogeneity was detected between the studies (I^2^ = 0%, p = 0.67), a fixed-effects model was applied to assess the data. The results from the meta-analyses showed that the risk of HT was not associated with contralateral occult PTC (pooled OR = 0.89, 95% CI = 0.61–1.30, p = 0.54) (**Figure 3D**).

### Contralateral Benign Nodule

Three studies estimated the effect of a contralateral benign nodule on contralateral occult PTC. Findings from the pooled analysis suggested that compared to patients without a contralateral benign nodule, patients with a contralateral benign nodule had a higher prevalence of contralateral occult PTC (pooled OR = 2.45, 95% CI = 1.20–5.03, p = 0.01) (**Figure 3E**).

### Lymph Node Metastasis

In our new data results, there were 65 (31.9%) patients who had central lymph node metastasis, 40 (19.6%) patients had lateral lymph node metastasis, and 99 (48.5%) patients without lymph node metastasis. However, in the contralateral occult PTC group, there were 14 (51.9%) patients with CLNM, 8 (29.6) patients with LLNM, and 5 (18.5%) patients without LNM. The results of the univariate analysis showed that the presence of contralateral occult PTC had a significant association with LNM (p<0.05). Also, the multivariable analysis showed that the presence of CLNM (OR = 2.086, 95% CI = 1.246–3.495, p=0.005) and LLNM (OR = 6.765, 95% CI = 1.877–24.380, p=0.003) were independent predictors of contralateral occult PTC (**Table 1**).

**Figure 4:**
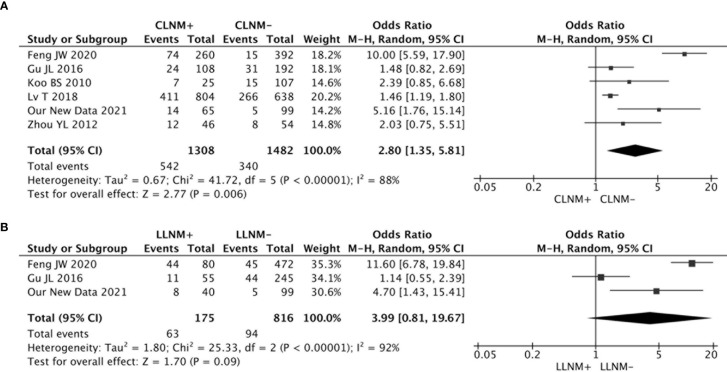
**(A)** The risk of contralateral occult PTC was significantly higher in patients with CLNM than patients without CLNM (pooled OR = 2.80, 95% CI = 1.35–5.81, p = 0.006); **(B)** There was no significant association between LLNM and contralateral occult PTC (pooled OR = 3.99, 95% CI = 0.81–19.67, p = 0.09).

An association between the risk of contralateral occult PTC and CLNM was investigated in six studies. The heterogeneity was high (I2 = 88%, p < 0.00001), and the results from the meta-analyses showed that the risk of contralateral occult PTC was significantly higher in patients with CLNM than patients without CLNM (pooled OR = 2.80, 95% CI = 1.35–5.81, p = 0.006) (**Figure 4A**).

There were three studies that analyzed the impact of LLNM on contralateral occult PTC. The analysis suggested no significant association between LLNM and contralateral occult PTC (pooled OR = 3.99, 95% CI = 0.81–19.67, p = 0.09) (**Figure 4B**).

## Discussion

Thyroid cancer has been the most rapidly increasing cancer over the past few decades in many countries. PTC comprises 90% of all thyroid malignancies and has an excellent prognosis with conventional therapies, such as surgery and radioactive iodine therapy. According to the latest ATA guidelines, TT is recommended for patients with a tumor > 4 cm, gross maximal ETE (T4), cervical lymph node metastasis, or distant metastasis. In addition, TL is endorsed as an initial surgical approach for low-risk, small- to medium-sized (T1-T2), N0 PTC in the absence of ETE (10). Considering that TT is associated with an increased risk of complications, such as vocal cord palsy and hypoparathyroidism (3, 21), TL may be sufficient for unilateral PTC with tumors < 4 cm and without ETE or clinical evidence of lymph node metastasis. There is no disagreement to performing TT for tumors detected preoperatively in bilateral lobes. However, when unilateral lobectomy is performed for patients with contralateral occult PTC, occult foci would lead to local recurrence and cervical lymph node metastasis and even re-operation, which is related to higher surgical risks compared with primary surgery (22, 23). Hence, we attempted to identify the risk factors for contralateral occult carcinoma to help select high-risk patients with occult carcinoma who would benefit from more extensive treatment.

Among patients with PTC, male patients have higher rates of ETE, regional lymph node, and distant metastasis, and mortality than female patients, regardless of age (24). Liu et al. reported that the male gender is an independent poor prognostic factor for all PTCs, and more aggressive treatment options should be considered for men (25). However, our pooled data showed that sex is not associated with the occurrence of contralateral occult PTC. Thus, there is no need to consider expanding the scope of lymph node dissection due to sex differences.

The biology of PTC is highly dependent on age, with young patients outperforming older patients in terms of survival (26). The previous guideline for thyroid cancer used 45 years as the cutoff age, and 55 years was the cutoff age used since 2017 (27). Thus, inclusion of a few of the literatures that used the previous guideline standard was inevitable. We set up two forest figures to suit two different cutoff values. Considering the effect of age on PTC’s biological activity, older patients whose PTC might more malignant, contralateral occult PTC might more likely to happen. However, the results of meta-analysis showed that regardless of whether the age cutoff value is 45 or 55 years, it did not affect the occurrence of the contralateral occult PTC. However, there were only two studies (included in our new data) that regarded 55 years as the cutoff age, and the OR value was higher than the 45 years cutoff age. Therefore, after expanding the sample size, this difference may become more significant.

Tumor size is the first factor evaluated for the biological characteristics of PTC because it is easily measured by preoperative US. The relationship between the size of the tumor and the presence of contralateral occult carcinoma remains controversial. It was concluded that tumor size is an independent predictor of bilaterality in PTC patients (28, 29). This is consistent with our new results. However, Pitt et al. examined a cohort of 228 patients and reported a rate of contralateral PTC for patients with primary tumors ≥1, <1, and <0.5 cm (30%, 26%, and 27%, respectively; nonsignificant p-value for all) (30). Combining the results of our meta-analysis, we suspected that Pitt’s results are limited by sample size. We suggest that researchers should perform a larger cohort study to clarify this problem.

Multifocality is a unique feature of PTC, and the rate of multifocality is reported to be 20% to 30% (31, 32). It was reported that increased tumor number is associated with higher rates of capsular invasion, ETE, and lymph node metastasis (33). A recent meta-analysis showed that multifocality in thyroid cancer increases the risk of disease recurrence (hazard ratio = 2.81; 95% CI = 1.07–7.36; p < 0.001) (34). What is more, Yoon et al. identified that multifocal tumors in the unilateral lobe have a high risk of bilateral carcinoma (9). Therefore, aggressive treatment, such as total thyroidectomy, central neck dissection, or postoperative radioactive iodine ablation, is suggested as the adequate treatment for multifocal PTCs. Our meta-analysis showed that patients with ipsilateral multifocal tumors have a 5.6-fold more contralateral occult PTC than patients with single-tumor foci. Thus, we strongly recommend patients with multifocality to pay attention to the presence of contralateral occult PTC.

An ETE is defined as an extension of the primary tumor beyond the thyroid capsule into the perithyroidal soft tissues, strap muscles, and adjacent structures (35). ETE is a well-known and significant factor for poor prognosis. It was reported that patients with ETE have a lower 10-year survival rate (75.2%) and a lower 10-year disease-specific survival rate (84.2%) (36). Also, Feng et al. reported that ETE is more commonly found in bilateral PTC than unilateral PTC (20.2% *vs*. 11.4%, p = 0.016) (37). Therefore, it is conjectured that ETE would be a risk factor for contralateral concealed PTC, and our retrospective research also got the same results. However, this was not the case. The meta-analysis results showed that there was no association between ETE and contralateral occult PTC. We considered that the reason is that the ETE (+) patients’ number was insufficient, ETE (+) patients accounted for about 7% to 37% of the total number in every study. This led to greater heterogeneity. What is more, the two studies with OR value <1 (from Gu et al. and Wang et al. respectively) had a percentage <10%. Less ETE (+) patients included, greater heterogeneity was important factor for the effect of ETE to contralateral occult PTC. Therefore, we suggest that researchers should perform a more perfect cohort study to get more credible conclusions.

In encapsulated thyroid neoplasms, the presence of capsular invasion is the most important criterion for separating benign neoplasms from malignancy. Currently, capsular invasion is defined in major guidelines as complete penetration of tumor capsules by neoplastic cells (38). Capsular invasion is not rare and has been reported from 9.9% to 26.8% of PTC (39). It was reported that capsular invasion is more common in patients with multifocal PTC and is related to tumor recurrence (33, 40). In our meta-analysis results, there is no connection between capsular invasion and contralateral occult PTC.

*BRAF* is a cytoplasmic protein kinase, and it is the main subtype of RAF kinase that could trigger tumorigenesis through the activation of the MAPK pathway. PTC patients with a *BRAF* mutation are associated with a higher risk of unfavorable clinicopathological characteristics (41). A study of 2,048 patients showed that a *BRAF* mutation is an independent risk factor for bilateral multifocal PTC (OR = 1.233, 95% CI = 1.063–1.431, p = 0.006) (42). However, we found no association between *BRAF* mutation and contralateral occult PTC. This may be because of the coexistent benign nodules in the contralateral lobe that could have affected the observed rate of contralateral occult carcinoma. The more useful reason may be the limited sample size; we considered that if there is an expanded sample size, the results would be reversed.

HT is the most common autoimmune thyroid disease. It causes the immune system to attack and destroy the thyroid gland and is the most common cause of primary hypothyroidism and nonendemic goiter. Liang et al. reported that PTC patients with HT have a higher rate of multifocality (p = 0.024) than patients without HT (42). This meta-analysis results showed no relation between HT and contralateral occult PTC.

Among the patients with unilateral PTC, a portion of them had contralateral thyroid nodules. A study of 1,442 patients reported that 667 (46.95%) patients with contralateral benign nodules were ultimately confirmed to be PTC (19). The therapeutic strategy for patients in this situation is controversial and challenging. Our data demonstrate that patients with contralateral benign nodules have increased contralateral occult PTC. This result may be because some small foci masked by a benign nodule in the contralateral lobe could lead to incorrect assessment during preoperative US or FNA. Therefore, we suggest that surgeons should evaluate the huge possibility of the presence of contralateral occult carcinoma in PTC patients with contralateral thyroid nodules and to deal with it more actively.

Multifocality might occur through the spread of an original tumor *via* intraglandular lymphatics. Cancer cells can cause metastasis to the contralateral gland through lymphatic dissemination, as well as lymph node metastasis. It is well established that PTC has a strong propensity for lymph node metastasis. Lymph node metastasis in PTC follows a predictable pattern. Most commonly, tumor cells metastasize to the central lymph node, followed by those of the lateral lymph node. Rarely, some patients develop LLNM in PTC without CLNM (43). It was reported that multifocality increases the risk of CLNM (44). However, the role of multifocality in LLNM remains a topic of debate (43, 45). Our results showed that patients with CLNM have more contralateral occult PTC but no significant relationship between LLNM and contralateral occult PTC. This might be because only three studies evaluated LLNM, and we considered that the result would be reversed by bringing in a large sample size research.

There are some limitations to this study. First, all of the studies were conducted in Asian countries. Second, most of the pooled studies were not prospective or randomized case-control trials. Third, the number of included studies was limited, which might have affected the results of our study, especially the risk factor analysis.

## Conclusion

The meta-analysis demonstrates the following risk factors for contralateral occult PTC: tumor size > 1 cm, ipsilateral multifocality, contralateral benign nodule, and CLNM. Sex, age, ETE, capsular invasion, *BRAF* mutation, HT, and LLNM do not affect contralateral occult PTC. Overall, although TT presents complications, the operation should be evaluated and implemented more actively when ≥ 1 risk factor is found in patients with PTC.

## Data Availability Statement

The original contributions presented in the study are included in the article/supplementary material. Further inquiries can be directed to the corresponding author.

## Ethics Statement

The studies involving human participants were reviewed and approved by Medical Science Research Ethics Committee of the First Hospital of China Medical University. Written informed consent for participation was not required for this study in accordance with the national legislation and the institutional requirements.

## Author Contributions

FZ is the first author of this study. WT is the corresponding author supervising this work. FZ, BZ, and XW collected the clinical medical records. FZ and BZ conceived and designed the meta-analysis. BZ and XY performed the meta-analysis. FZ performed the statistical analyses of all the data and wrote the manuscript. XW and SW contributed material/analysis tools. FZ and BZ collected reference and managed the data. FZ and WT designed the study. All authors contributed to the article and approved the submitted version.

## Funding

This study was supported by The Research Fund for Public Welfare, National Health and Family Planning Commission of China (grant 201402005). The funder had no role in study design, data collection or analysis, or in the presentation or publication of the results.

## Conflict of Interest

The authors declare that the research was conducted in the absence of any commercial or financial relationships that could be construed as a potential conflict of interest.

## Abbreviations

PTC, papillary thyroid carcinoma; CLNM, central lymph node metastasis; ETE, extrathyroidal extension; HT, Hashimoto thyroiditis; LLNM, lateral lymph node metastasis; TC, thyroid carcinoma; PTMC, papillary thyroid microcarcinoma; WHO, World Health Organization; US, neck ultrasound; FNA, fine-needle aspiration; TL, thyroid lobectomy; TT, total thyroidectomy; SD, standard deviations; CIs, confidence intervals.
